# The impact of a Western diet and binge drinking on metabolic dysfunction‐associated steatotic liver disease in male and female mice

**DOI:** 10.1113/EP093502

**Published:** 2026-02-17

**Authors:** Ihsan Shawki Akili, Corina Miko, Azhar Saeed, Colby Filosa, Madeline Orlowski, Alanna Sherman, Aldi Chan, Madison Wan, Jake Grenon, Alec Thibeault, Catherine Young, Melinda Wetzel, Virginia Clubb, Matthew A. Caporizzo, R. Chris Skinner

**Affiliations:** ^1^ Department Nutrition and Food Sciences University of Vermont Burlington Vermont USA; ^2^ Animal Biosciences Program University of Vermont Burlington Vermont USA; ^3^ Department of Pathology and Laboratory Medicine University of Vermont Burlington Vermont USA; ^4^ Larner College of Medicine University of Vermont Burlington Vermont USA; ^5^ Department of Biology Landmark College Putney Vermont USA; ^6^ Neuroscience Program University of Vermont Burlington Vermont USA; ^7^ Department of Rehabilitation and Movement Sciences University of Vermont Burlington Vermont USA; ^8^ Department of Molecular Physiology and Biophysics University of Vermont Burlington Vermont USA

**Keywords:** alcohol, binge drinking, liver disease, metabolic dysfunction‐associated steatohepatitis, metabolic dysfunction‐associated steatotic liver disease, nutrition, sex differences, Western diet

## Abstract

Metabolic dysfunction‐associated steatotic liver disease (MASLD) is a major public health concern, largely driven by the high‐fat, high‐sugar and low‐fibre Western diet. The Western diet is often accompanied by binge drinking, or the rapid consumption of alcohol in a short time leading to a blood alcohol concentration of ≥0.08%. Emerging evidence suggests that co‐consumption of a Western diet and binge drinking might converge on shared pathological mechanisms in males, but little is known about the role of sex in this interaction. Given these complex interactions between diet, alcohol, sex and liver health, we sought to investigate how the addition of binge drinking to a Western diet impacts MASLD progression in male and female mice. Young (6‐week‐old) male and female C57BL6/J mice (*n* = 9–11 per sex per group) were assigned to the following groups: Control, Control/Binge, Western, or Western/Binge. Following 12 weeks of treatment, Western diet, regardless of binge drinking, resulted in increased hepatic lipid accumulation and oxidative damage in both sexes, with males showing more evidence of disease than females. In general, male mice in the Western binge group had the most pronounced evidence of liver damage among males, whereas female mice in the Western diet group tended to have more evidence of disease than all other female groups as measured by histology, serum transaminases and immunohistochemistry. The collective results suggest that addition of binge drinking to a Western diet can result in a more progressive form of liver disease in males and that sex plays a key role in response to the combination of unhealthy diet and binge drinking.

## Abstract (highlights)

- **What is the central question of this study?**
  Does addition of binge drinking to a Western diet result in a more progressive liver disease than either alone, and how is this modulated by sex?
- **What is the main finding and its importance?**
  In male mice, combining binge drinking and a Western diet resulted in more pronounced detriments in key liver measures than either factor alone, whereas females showed greater liver damage in response to a Western diet alone than to the combined exposure.

## 1 INTRODUCTION

Metabolic dysfunction‐associated steatotic liver disease (MASLD) is a major public health concern affecting approximately one‐third of the US population (J. Li et al., 2025). It is driven primarily by the Western diet, a diet pattern typified by high amounts of saturated and *n*‐6 fats and simple sugars paired with low amounts of *n*‐3 fats and dietary fibre (Huang et al., 2025). Associated with nearly every cardiometabolic disease, the Western diet was initially named owing to its prominence in North America and Europe, but it has become widespread in the past few decades, resulting in significant increases to the global burden of liver diseases (Huang et al., 2025; Yang et al., 2024). Although MASLD is driven primarily by diet, alcohol contributes to worsening liver health, and those who consume an unhealthy diet often consume varying amounts alcohol as well (Hagström, 2017; Liu et al., 2025). Although chronic drinking has traditionally been a major concern, binge drinking, or drinking resulting in a blood alcohol concentration of ≥0.08%, corresponding to roughly 4+ drinks in females and 5+ drinks in males within 2 h, has recently become the most common form of immoderate alcohol consumption, with many individuals regularly participating in binge drinking throughout young adulthood and beyond (Bohm, 2021; Kanny et al., 2020; Understanding Binge Drinking | National Institute on Alcohol Abuse and Alcoholism (NIAAA), n.d.).

Previous research on the combination of unhealthy diet and alcohol consumption suggests potential for their combination to converge on linked pathways that drive progressive liver injury through excessive hepatic fat accumulation and a pro‐inflammatory response (Y. Li et al., 2024; Osna et al., 2022). Overconsumption of sugars, fats and alcohol all upregulate *de novo* lipogenesis and impair β‐oxidation, resulting in heightened triglyceride storage (Ameer et al., 2014; Osna et al., 2022). Over time, build‐up of lipotoxic substances associated with this increased fat storage can drive increased pro‐inflammatory cytokine signalling and immune infiltration (Duan et al., 2022; Rafaqat et al., 2024; H. J. Wang et al., 2012). This can result in sustained hepatocyte injury and systemic inflammation leading to more progressive liver disease, such as metabolic dysfunction associated steatotic liver disease (MASH) and fibrosis (Akkız et al., 2024). Most research on the combination of unhealthy diet and drinking on liver health has focused on chronic drinking, with little research on binge drinking and no studies on the role of sex in this relationship (Bohm, 2021; Ji & Cheng, 2023). This is significant, because major sex differences in liver disease exist, with males being more susceptible to diet‐driven liver disease, whereas females are more prone to alcohol‐induced liver damage (Ballestri et al., 2017; Lonardo et al., 2019; Seidemann et al., 2024). Given the complex intersections between unhealthy diet, binge drinking, sex and liver disease, we sought to investigate whether the addition of binge drinking to a Western diet would result in a more severe form of MASLD in male and female mice.

## 2 MATERIALS AND METHODS

### 2.1 Ethical approval

All animal procedures were approved by the Animal Care and Use Committee at the University of Vermont (UVM) (Protocol #X3‐053), and all experiments were conducted in accordance with the National Research Council for the Care of Laboratory Animals.

### 2.2 Animals, diets and binge drinking

Young (6‐week‐old) male and female C57BL6/J mice (*n* = 9–11 per sex per group) were purchased from Jackson Laboratories (Bar Harbor, ME, USA). Mice were kept in the UVM Animal Care Facility in a temperature‐ and humidity‐controlled room on a 12 h–12 h light–dark cycle. Mice were maintained on a reverse light cycle to allow for more accurate measurements of food and alcohol consumption. This trial was performed in batches, with the initial batch having mice (*n* = 6 or 7 per sex per group) single housed to collect important food and liquid consumption data and these tissues being used for most experiments. A subsequent batch included co‐housed mice (*n* = 3 or 4 mice per cage), used to perform follow‐up experiments. Owing to the nature of these experiments, researchers were not blinded to treatment groups; however, in subsequent analyses of tissues blinding was implemented to reduce bias.

In all batches, following a week of acclimation mice were randomly assigned to one of four groups, termed Control, Control/Binge, Western or Western/Binge. Control mice consumed the AIN‐93G diet, whereas Western mice consumed a custom diet formulated to mimic the typical American diet in collaboration with Dr Lauren Brizgys (Inotiv, West Lafeyette, IN, USA). Briefly, this diet featured high amounts of sucrose, fructose, saturated fatty acids and *n*‐6 fatty acids, with low amounts of *n*‐3 fatty acids and dietary fibre. Detailed information on diets can be found in Table 1. Mice were provided with ad libitum access to food throughout the experiment, with food consumption being measured weekly and food being refreshed during this measurement. The feed conversion ratio (FCR) was calculated by dividing the total food consumed divided by final body weight. Mice were provided with ad libitum access to distilled water throughout the study. Mice consuming alcohol followed a modified version of the Drinking in the Dark protocol (Thiele & Navarro, 2014). Briefly, ∼2–3 h into their dark cycle, mice were provided with a bottle of 20% v/v ethanol (Pharmco, Brookefield, CT, USA). Mice were allowed access to this bottle for 3–6 h for 3 days per week, with 1 or 2 days in between each binge drinking session (Monday, Wednesday and Friday). At the start and end of each binge drinking session, the liquid was weighed to determine consumption.

**TABLE 1:** Nutrient composition of diets used in the study.

Nutrient	Control^a^	Western
Carbohydrates (%)	64	42
Simple sugars (%)	10	30
Dietary fibre (%)	5	2.5
Fat (%)	17	44
Saturated fats (%)	1	28
*n*‐6:*n*‐3 ratio	5:1	17:1
Protein (%)	17	15

Abbreviations: AIN, American Institute of Nutrition.

^a^ Control diet is AIN 93‐G.

At the conclusion of the study, mice were fasted for ∼6 h and euthanized via inhalation of isoflurane (3%–5%) and cardiac exsanguination. Organs were immediately collected, weighed and preserved. At the conclusion of the study, one mouse from the male Western/Binge group was excluded from analyses because it regularly did not consume alcohol and consumed significantly less food than all other mice, and one mouse from the female Control/Binge group was excluded because it had to be euthanized prior to the end of the study.

### 2.3 Liver assays

Hepatic triglyceride content was quantified using a commercially available colorimetric assay kit (Cayman Chemical, Ann Arbor, MI, USA). Liver tissue (150–200 mg) was homogenized in 1 mL of diluted NP‐40 assay buffer, prepared using the standard diluent provided with the kit. The homogenate was then centrifuged at 10 000*g* for 10 min at 4°C, and the resulting supernatant was collected and diluted further at a 1:5 ratio with the same assay diluent. Aliquots of the diluted supernatant (10 µL) were added to a 96‐well plate along with 150 µL of the provided lipoprotein lipase enzyme solution. The plate was incubated at room temperature for 60 min, and absorbance was measured at 540 nm using a BioTek Epoch microplate spectrophotometer (Winooski, VT, USA). All samples were assayed in duplicate.

Hepatic lipid peroxidation was quantified using a commercially available colorimetric Thiobarbituric acid reactive substances (TBARS) assay kit, based on the trichloroacetic acid (TCA) precipitation method (Cayman Chemical). Approximately 25 mg of liver tissue was homogenized in 250 µL of diluted RIPA buffer. The homogenate was centrifuged at 1600*g* for 10 min at 4°C. Subsequently, 100 µL of the supernatant was mixed with 100 µL of the TCA reagent and incubated in boiling water for 1 h to initiate the reaction. The reaction was then stopped by placing the samples on ice, followed by centrifugation at 1600*g* for 10 min at 4°C. Finally, 200 µL of the resulting supernatant was transferred to a 96‐well plate, and absorbance was measured at 540 nm using a BioTek Epoch microplate spectrophotometer. All samples were analysed in duplicate.

Hepatic myeloperoxidase (MPO) as a marker of inflammation and leucocyte infiltration was quantified using a commercially available colorimetric assay kit (Invitrogen, Thermo Fisher Scientific, Waltham, MA, USA), following the manufacturer's instructions. Briefly, 25–40 mg of liver tissue was homogenized in 760 µL of the assay kit's standard diluent. The resulting homogenate was centrifuged, and the supernatant was diluted 1:1. A 20 µL aliquot of the diluted supernatant was mixed with 20 µL of the provided clarifying reagent in a microcentrifuge tube and incubated at 37°C for 30 min. Following this, 5 µL of the acid reagent was added, and the mixture was incubated at 60°C for 10 min. After incubation, the samples were centrifuged at 3000*g* for 10 min. A 300 µL aliquot of the final supernatant was transferred to a 96‐well plate, and absorbance was measured at 460 nm using a BioTek Epoch microplate spectrophotometer. All samples were run in duplicate.

Hepatic reduced glutathione (GSH) was measured using a commercially available colorimetric assay kit (Invitrogen), according to the manufacturer's protocol. Briefly, 25–40 mg of liver tissue was homogenized in 270 µL of PBS (0.01 M, pH 7.4) at 4°C. The homogenate was then centrifuged at 10 000*g* for 10 min at 4°C, and the resulting supernatant was diluted 1:1 with the same PBS solution. A 100 µL aliquot of the diluted sample was mixed with 100 µL of the kit‐provided acid reagent in a microcentrifuge tube and centrifuged again at 4500*g* for 10 min. Subsequently, 100 µL of the resulting supernatant was transferred to a 96‐well plate and mixed with 25 µL of 5,5′‐dithiobis‐(2‐nitrobenzoic acid) (DTNB) reagent, followed by 100 µL of the phosphate buffer solution. The reaction was incubated at room temperature for 5 min. Absorbance was then measured at 405 nm using a BioTek Epoch microplate spectrophotometer. All samples were analysed in duplicate.

Likewise, hepatic superoxide dismutase (SOD) was measured using a commercially available colorimetric assay kit (Invitrogen), following the manufacturer's instructions. Briefly, 100 mg of liver tissue was homogenized in PBS and centrifuged at 1500*g* for 10 min at 4°C to retain both cytosolic (SOD1) and mitochondrial (SOD2) isoforms in the supernatant. The resulting supernatant was diluted 1:4 with the assay buffer provided in the kit. A 10 µL aliquot of the diluted sample was transferred to a 96‐well plate and mixed with 50 µL of the 1× substrate solution and 25 µL of the 1× xanthine oxidase solution (both provided by the kit). The reaction mixture was incubated at room temperature for 20 min. Absorbance was then measured at 450 nm using a BioTek Epoch microplate spectrophotometer. All samples were analysed in duplicate.

### 2.4 Histology and immunohistochemistry

A section of the left lobe of the liver was excised and immediately fixed in 10% neutral‐buffered formalin for histological evaluation. Fixed tissue samples were submitted to the Histology Laboratory of the University of Vermont Medical Center for paraffin embedding, sectioning (7 µm thickness), and Hematoxylin and Eosin or Masson's Trichrome staining. Histological evaluation was performed by Azhar Saeed, MD, a board‐certified gastrointestinal pathologist. Liver sections were assessed for the presence and extent of both macrovesicular and microvesicular steatosis. In addition to steatosis grading, other histopathological features indicative of steatohepatitis, including hepatocellular ballooning, lobular inflammation, portal inflammation and fibrosis, were evaluated.

The left half of the spleen and the right medial lobe of the liver were preserved in 10% neutral buffered formalin. The University of Vermont Medical Center Histology Laboratory fixed, sectioned, developed and mounted slides of each for key targets associated with immune infiltration. Spleens were sectioned at 5 µm thickness and livers at 7 µm. For the spleen, stains were performed for CD8, CD68 and C45, and for the liver CD8 and CD68 were performed. Immunohistochemistry slides were analysed by two researchers, blinded to their treatments, using a Nikon TE 2000‐S light microscope (Nikon Instruments, Melville, NY, USA) and ImageJ for quantification.

### 2.5 Serum chemistry measurements

Blood was collected and centrifuged at 1500*g* for 10 min at 4°C to separate serum, which was then stored at −80°C until further analysis. Biochemical assessments included glucose, total cholesterol, alanine aminotransferase (ALT), aspartate aminotransferase (AST), albumin, globulin and total thyroxine. All measurements were performed enzymatically using a commercially available VetScan V2 chemistry analyser with preloaded reagent rotors (Zoetis, Parsippany Hills, NJ, USA). Briefly, for each sample, 100 µL of serum was loaded into the rotor, which contained all necessary reagents for each individual assay, and processed automatically according to the manufacturer's instructions.

### 2.6 Statistical analysis

Differences in group means were assessed using two‐way ANOVA followed by Tukey's *post hoc* testing for multiple comparisons. A significance level of *P *< 0.05 was used to determine statistical significance. All statistical analyses and data visualizations were performed using GraphPad Prism version 10.5.0.

## 3 RESULTS

### 3.1 Weight and consumption patterns

**FIGURE 1:**
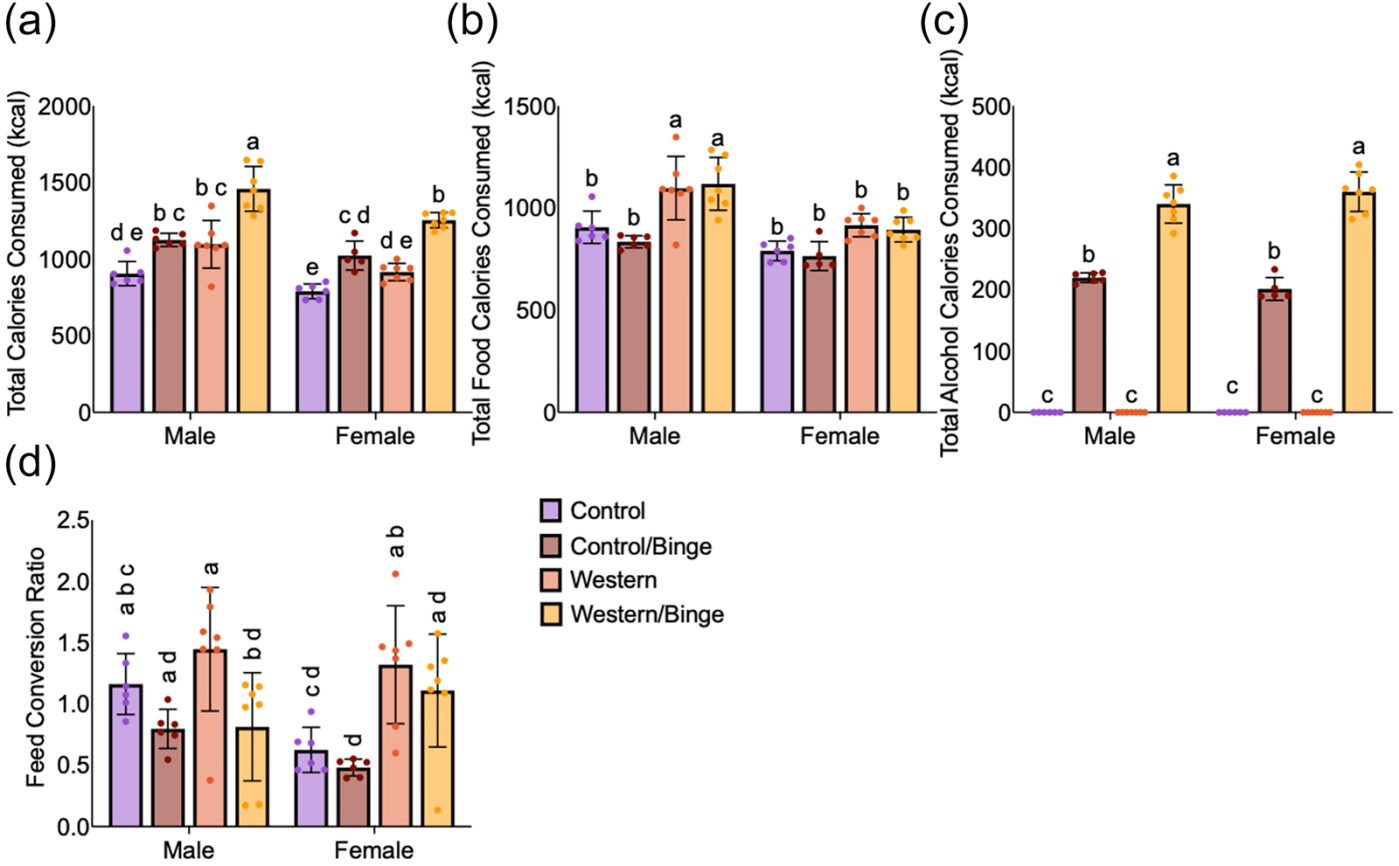
Consumption patterns of total calories (a); food calories (b) and alcohol calories (c), along with food conversion ratio (d) of mice consuming different diets paired with binge drinking. All data are shown as the mean ± SD, with *n *= 5–7 mice per group. Significance was determined at *P *< 0.05, and different letters indicate statistical differences.

All consumption data, in addition to adipose tissue and sex organ weights represented are only from single‐housed animals. Final weight, liver weight and spleen weight include data from both single‐ and group‐housed animals. Single‐ versus group‐housed statistical comparisons were made and no differences observed between matched groups between batches (Tables S1 and S2). The addition of binge drinking significantly increased total calorie consumption in Control/Binge mice and Western/Binge mice regardless of sex (*P *< 0.0001) compared with their respective diet groups (Figure 1a). Male Western (*P *= 0.010) and Western/Binge (*P *= 0.003) mice had an increase in food calorie consumption compared with male Control and Control/Binge mice; however, this pattern was not present in female mice (Figure 1b). The alcohol calorie contribution was greater (*P *< 0.0001) for Western/Binge compared with Control/Binge regardless of sex (Figure 1c). Male Western/Binge mice had lower (*P *= 0.047) FCR compared with male Western mice. Although not significant, male Control/Binge mice also had a noticeable reduction in FCR. This same reduction was not observed in female Western/Binge mice; however, female Western mice had a higher FCR compared with female Control (*P *= 0.030) and Control/Binge (*P *= 0.004) mice (Figure 1d).

**FIGURE 2:**
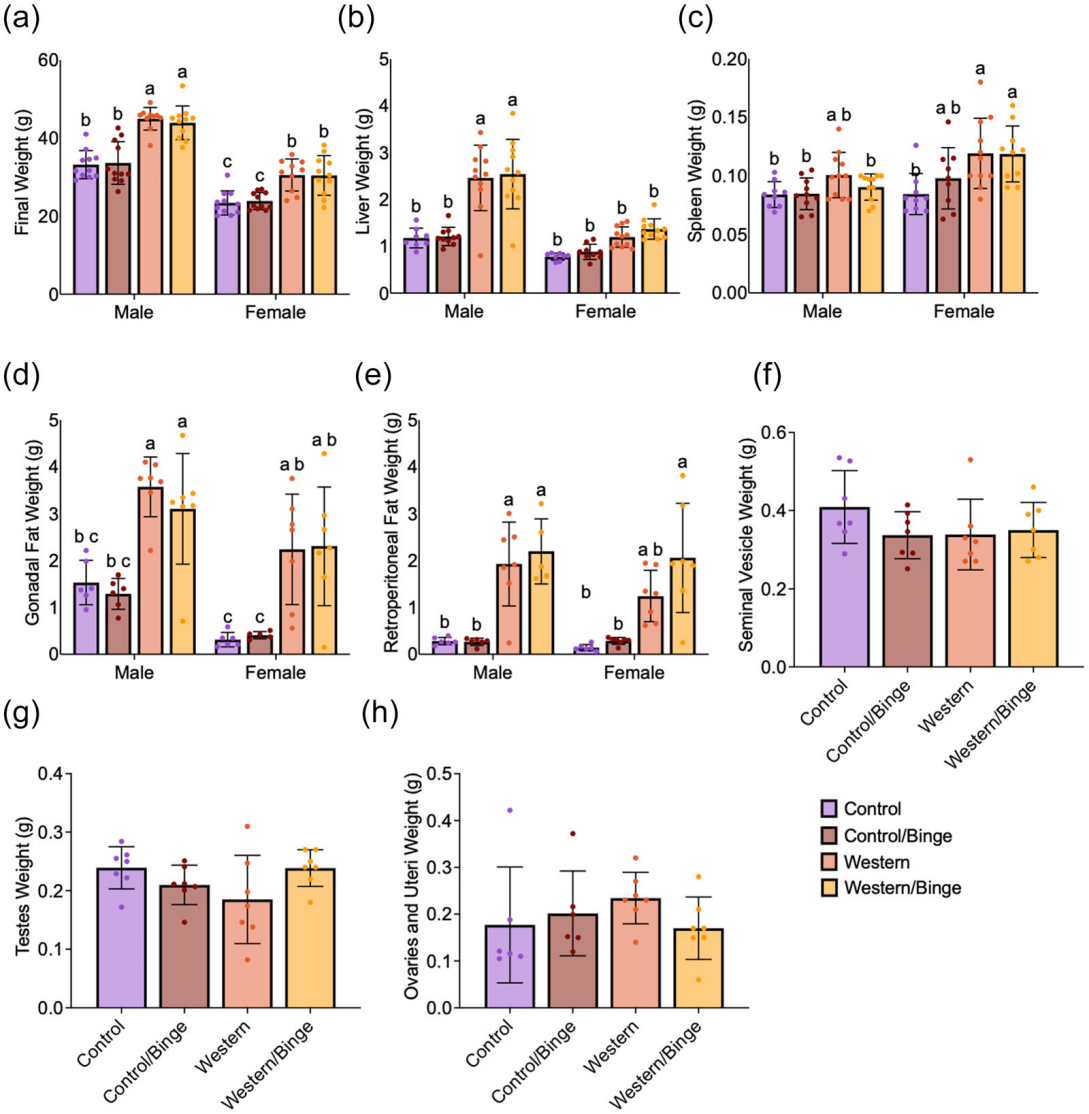
Weight measures of the body (a), liver (b), spleen (c), gonadal fat (d), retroperitoneal fat (e), seminal vesicles (f), testes (g) and ovaries and uteri (h) in mice consuming different diets with and without binge drinking. All data shown as the mean ± SD, with *n* = 5–11 mice per group. Significance was determined at *P *< 0.05, and different letters indicate statistical differences.

At baseline, there were no significant differences in body weight among the experimental groups (data not shown). However, after 12 weeks, Western mice exhibited a statistically significant increase in body weight compared with male Control (*P *< 0.0001), male Control/Binge (*P *< 0.0001) and Female Control (*P *< 0.0001). Binge drinking did not significantly increase weight compared with the respective diet‐only groups. Female Western and Western/Binge mice had an increase in body weight that was non‐significant compared with Control and Control/Binge males (Figure 2a). Male Western mice, regardless of binge drinking, had significantly heavier (*P *< 0.0001) livers than all other groups. No differences in liver weight were observed in females among all groups, although female Western/Binge mice did have a trend (*P *= 0.060) towards heavier livers than female Control mice (Figure 2b). No differences were observed in the males for spleen weight, but female Western mice had significantly heavier spleens compared with female Control (*P* = 0.007). Female Western and Western/Binge mice had heavier spleens than male Control, Control/Binge and Western/Binge (*P *= 0.042) (Figure 2c). Western diet, in both males and females, increased (*P *= 0.008) gonadal adipose tissue weight compared with the respective Control and Control/Binge groups (Figure 2d). Western diet increased (*P *= 0.0002) retroperitoneal adipose tissue regardless of binge drinking in males compared with male Control and Control/Binge. In females, only Western/Binge increased (*P *< 0.0001) retroperitoneal adipose tissue to be different from female Control and Control/Binge, although female Western mice did have a trend (*P *= 0.054) for heavier retroperitoneal adipose compared with female Control.

### 3.2 Liver histology

**FIGURE 3:**
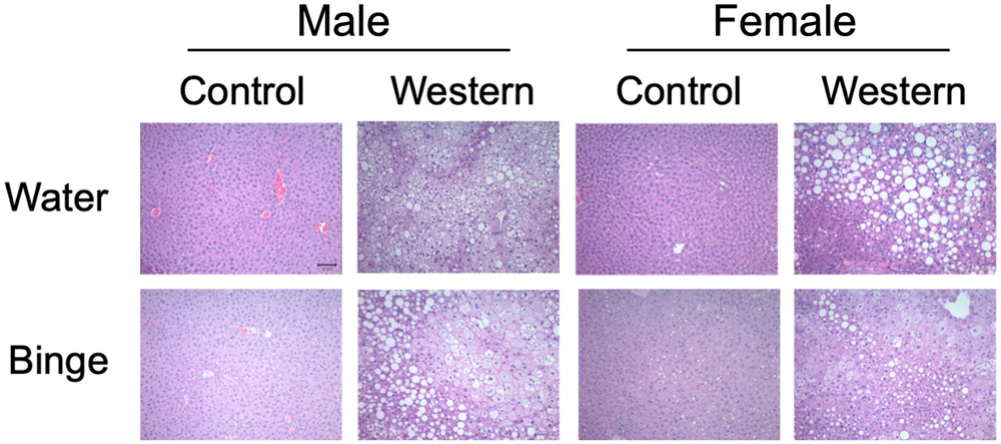
Representative images of liver histology in male and female mice consuming different diets with and without alcohol. All photographs were taken at 20× magnification. Scale bar: 25 µm.

Liver histology includes only single‐housed mice. Minimal steatosis was observed in male or female Control or Control/Binge groups. Western and Western/Binge mice, regardless of sex, had average steatosis scores of >80%. Female Western and Western/Binge mice were more likely to have microvesicular steatosis, whereas male Western and Western/Binge mice were more likely to have macrovesicular steatosis. Females were more likely to have a diffuse pattern of microvesicular steatosis, whereas males were more likely to show a pericentral pattern. No macro‐ or microvescicular steatosis was observed in any Control or Control/Binge mice regardless of sex. Male Western/Binge mice had the most lobular inflammation of all groups. Portal inflammation was not observed in any mice except one female Western. Trichrome staining did not reveal any evidence of fibrosis. Representative images can be found in Figure 3 and details in Table 2.

**TABLE 2:** Liver histology scoring for males and females consuming different diets with and without binge drinking.

	Male				Female			
Variable	Control	Control/Binge	Western	Western/Binge	Control	Control/Binge	Western	Western/Binge
Steatosis percentage average	<5	<5	71	90	0	<5	92	82
Macrovesicular, %	0	0	32	43	0	<5	38	23
Microvesicular, %	0	0	47	56	0	0	55	70
Pattern	N/A	N/A	Pericentral	Pericentral	N/A	N/A	Diffuse	Diffuse
Hepatocyte ballooning presence, %	0	0	0	17	0	0	14	17
Lobular inflammation presence, %	<5	<5	83	100	<5	<5	50	71
Portal inflammation presence, %	0	0	0	0	0	0	17	0
Fibrosis, %	0	0	0	0	0	0	0	0

*n* = 5–7 mice per group. [Correction added on 7 March 2026, after first online publication: Few values have been corrected in Table 2.]

### 3.3 Liver assays

**FIGURE 4:**
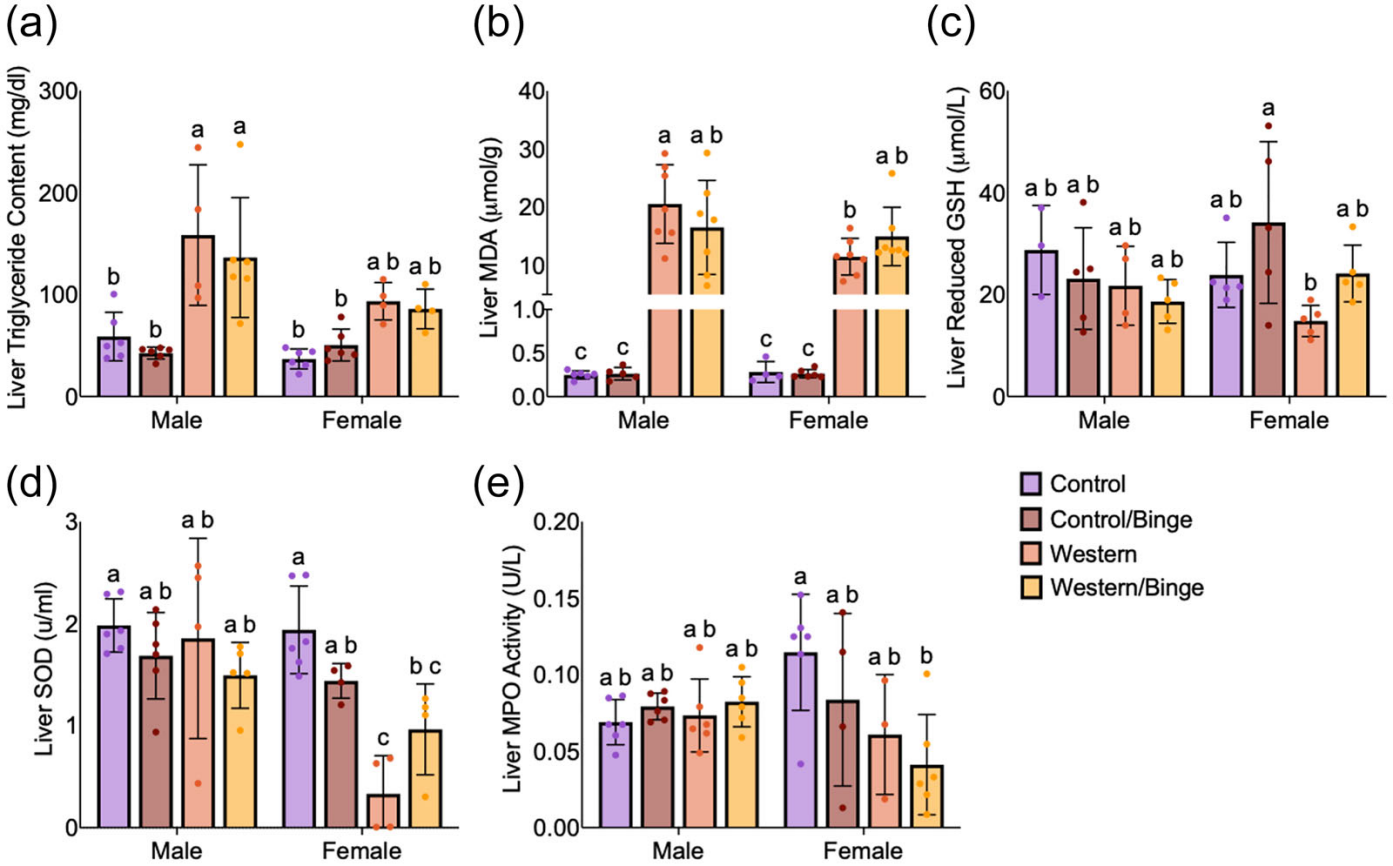
Hepatic measures of triglycerides (a), malonaldehyde (b), reduced glutathione (c), superoxide dismutase (d) and (e) myeloperoxidase in mice consuming different diets with and without binge drinking. All data are shown as the mean ± SD, with *n* = 3–7 per group. Significance was determined at *P *< 0.05, and different letters indicate statistical differences. Abbreviations: GSH, glutathione; MDA, malonaldehyde; MPO, myeloperoxidase; SOD, superoxide dismutase.

Liver assays include only single‐housed mice. Hepatic triglyceride content was significantly higher in both male Western (*P *= 0.007) and Western/Binge (*P = *0.001) mice compared with Control and Control/Binge. In females, hepatic triglyceride content was also elevated in Western and Western/Binge groups relative to Control and Control/Binge; however, these differences did not reach statistical significance. Binge drinking did not result in a difference in hepatic triglyceride content, regardless of diet (Figure 4a).

Hepatic malondialdehyde levels were significantly higher in both male Western (*P <* 0.0001) and Western/Binge (*P <* 0.0001) mice compared with male Control and Control/Binge. Likewise, in females, hepatic malondialdehyde levels were significantly elevated in the Western (*P = *0.008) and Western/Binge (*P = *0.0002) groups compared with female Control and Control/Binge. Male Western mice had significantly higher hepatic lipid peroxidation compared with female Western (*P *= 0.015), but no differences between male Western/Binge or female Western/Binge were observed. Binge drinking did not significantly increase hepatic lipid peroxidation compared with respective non‐binge counterparts in either sex (Figure 4b).

Hepatic reduced glutathione levels did not differ significantly in males. However, female Western mice had a significant reduction (*P *= 0.025) in reduced glutathione compared with female Control/Binge but not Control (Figure 4c). In males, hepatic total SOD was not significantly different between groups. However, in females, hepatic SOD levels were significantly lower in both Western (*P = *0.0002) and Western/Binge (*P *= 0.046) compared with female Control. Female Western hepatic SOD was significantly lower (*P *= 0.046) than all other groups, regardless of sex, except Western/Binge (Figure 4d). In males, liver MPO activity, a marker of neutrophil infiltration and hepatic inflammation, did not differ significantly among groups. In females, the Western/Binge group had significantly reduced (*P *= 0.031) MPO levels compared with female Control. Binge drinking did not significantly change MPO compared with the respective diet groups in males or females (Figure 4e).

### 3.4 Serum chemistry measures

**FIGURE 5:**
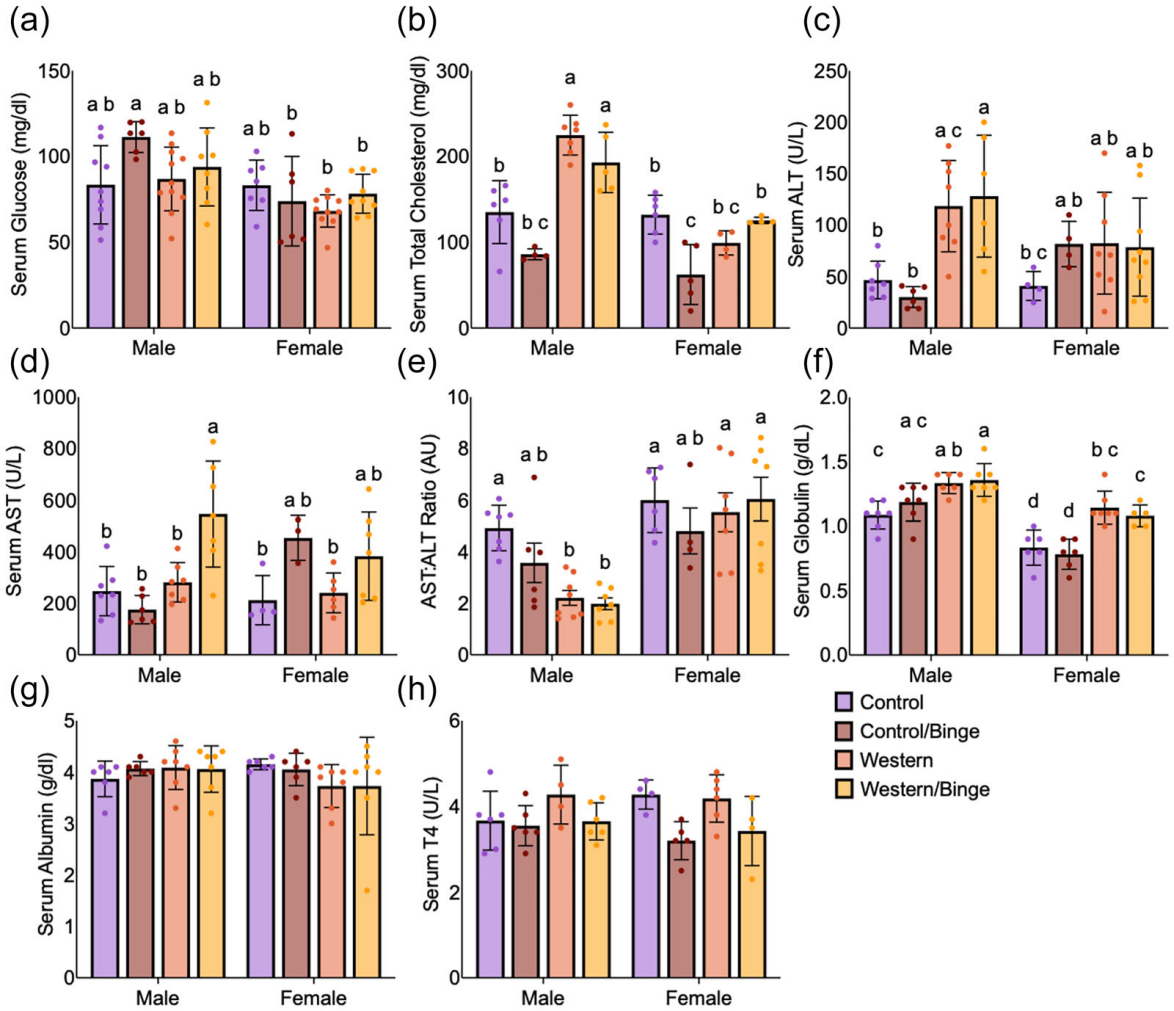
Serum measures of glucose (a), total cholesterol (b), ALT (c), AST (d), AST:ALT ratio (e), globulin (f), albumin (g) and thyroxine (h) in mice consuming different diets with and without binge drinking. All data are shown as the mean ± SD, with *n* = 3–7 per group. Significance was determined at *P *< 0.05, and different letters indicate statistical differences. Abbreviations: ALT, alanine aminotransferase; AST, aspartate aminotransferase; T_4_, thyroxine.

Serum measures include only single‐housed mice. Serum fasting glucose displayed no differences within sex, but male Control/Binge mice had higher glucose than female Control/Binge (*P *= 0.011), Western (*P *= 0.004) and Western/Binge (*P *= 0.017) but not Control (Figure 5a). Meanwhile, male Western and Western/Binge mice had increased (*P *< 0.0001) total cholesterol compared with all other groups. Female Control/Binge mice had reduced (*P *= 0.023) total cholesterol compared with both female Western/Binge and Control, but not Western mice (Figure 5b).

In males, serum ALT levels were significantly higher in the Western (*P *= 0.04) and Western/Binge (*P *= 0.015) mice compared with male Control and Control/Binge. Male Western/Binge mice had higher (*P *= 0.032) serum ALT only than female Control animals, with Western having a trend (*P *= 0.054) compared with female Control (Figure 5c). Male Western/Binge showed higher (*P *= 0.006) serum AST than all other male groups. Only female Western and Control had lower (*P *= 0.002) AST than male Western/Binge mice (Figure 5d). For AST:ALT ratio, male Western and Western Binge groups had lower (*P *= 0.015) ratios than male Control but not Control/Binge. Male Western and Western/Binge groups were not significantly different from female Control/Binge (Figure 5e). No differences within sex were observed for females.

Male Western (*P *= 0.012) and Western/Binge (*P *= 0.0002) mice had higher serum globulin compared with male Control. Meanwhile, female Western (*P *= 0.0008) and Western/Binge (*P *= 0.04) mice had higher serum globulin than female Control and Control/Binge. Across sexes, all male groups had higher (*P *< 0.0001) serum globulin than female Control and Control/Binge mice. Male Western (*P *= 0.022) and Western/Binge (*P *= 0.006) mice had higher serum globulin than female Western/Binge, but only male Western/Binge had higher (*P *= 0.033) serum globulin compared with female Western (Figure 5f). No differences were observed in serum albumin (Figure 5g) or serum thyroxine (Figure 5h).

### 3.5 Liver and spleen immunohistochemistry

**FIGURE 6:**
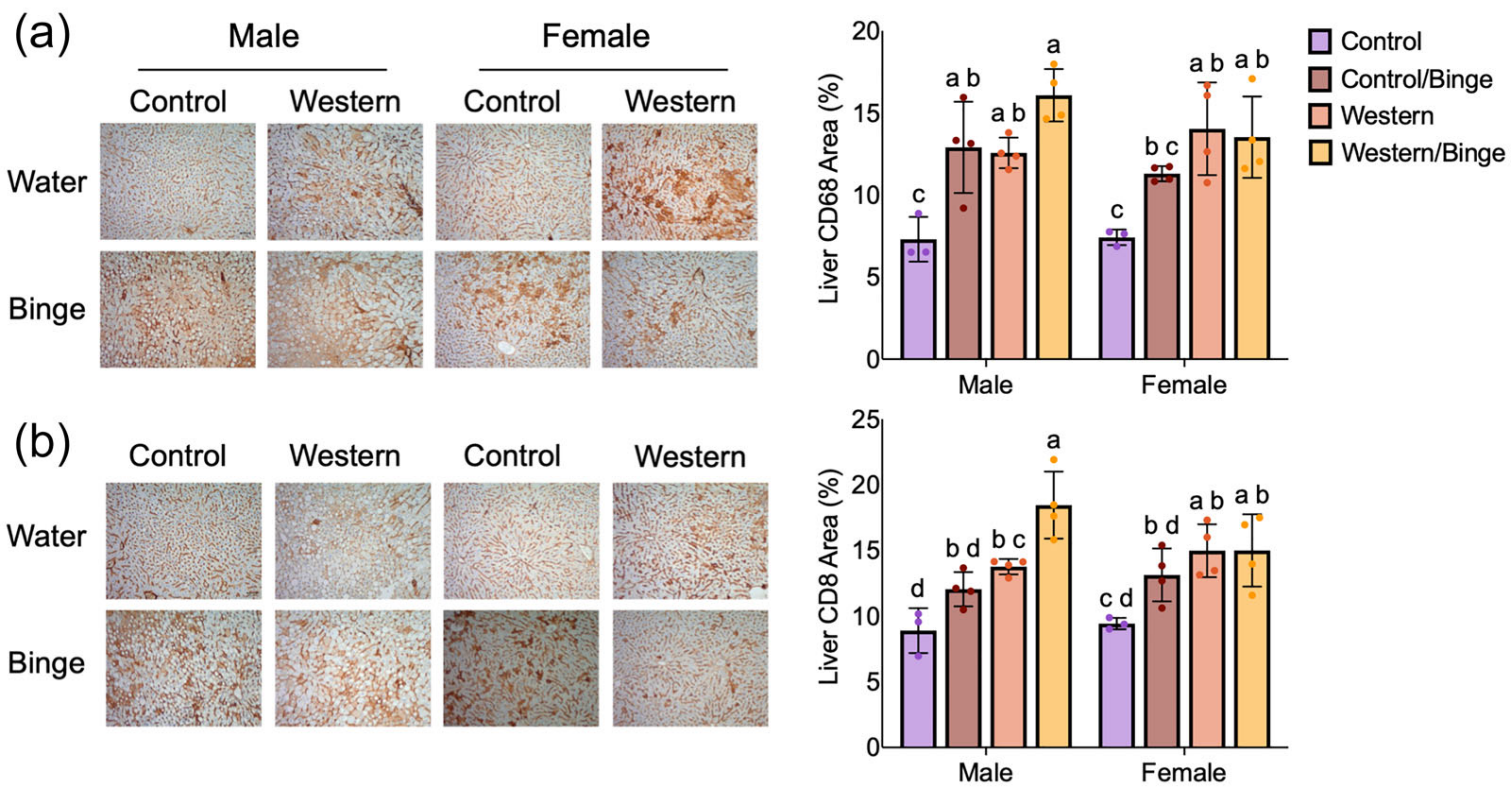
Representative images and quantifications for immunohistochemical staining of liver for CD68 (a) and CD8 (b). All data are shown as the mean ± SD, with *n* = 3 or 4 per group. Significance was determined at *P *< 0.05, and different letters indicate statistical differences. All photographs were taken at 20× magnification. Scale bar: 25 µm.

**FIGURE 7:**
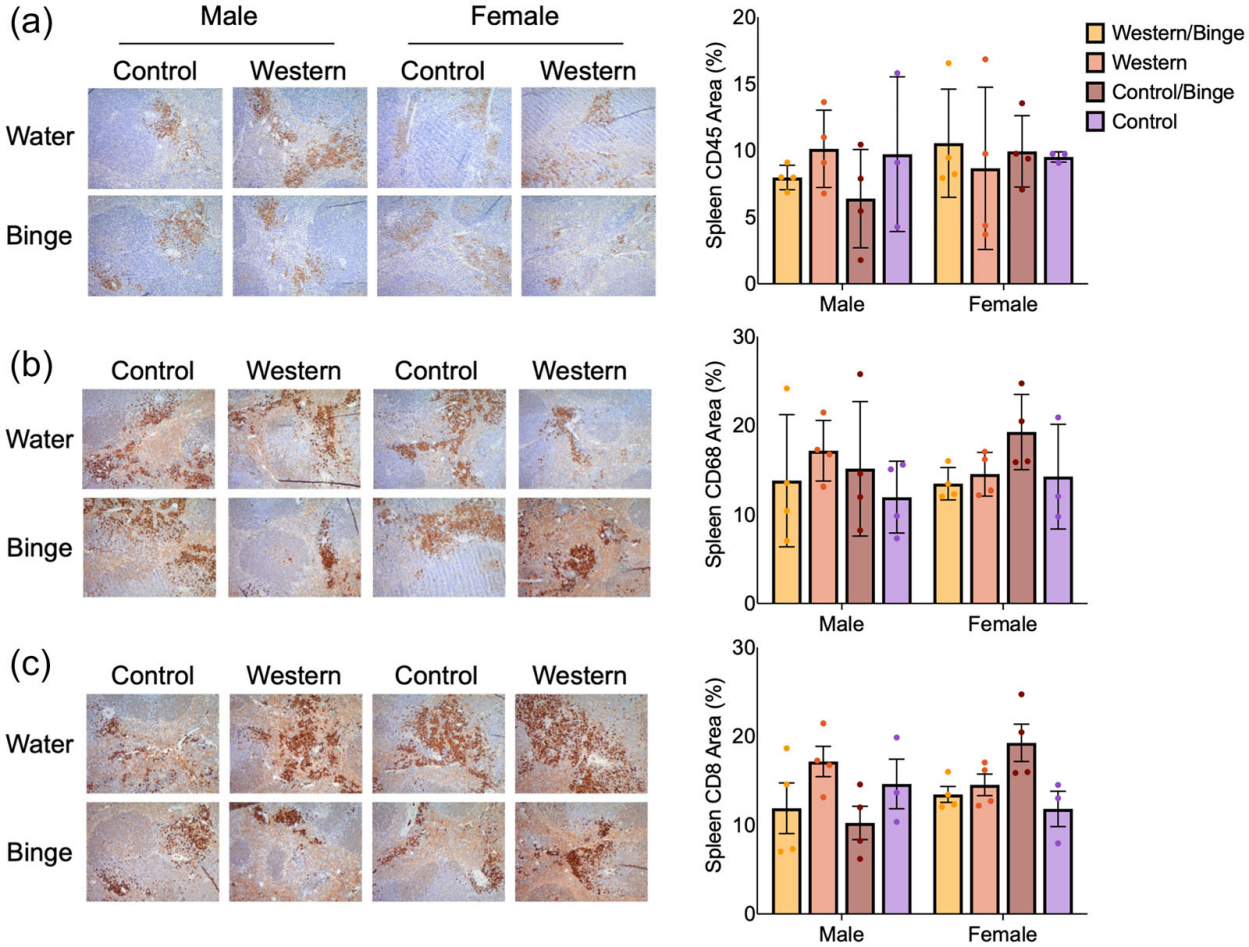
Representative images and quantifications for immunohistochemistry staining of spleens for CD45 (a), CD68 (b) and CD8 (c). All data are shown as the mean ± SD, with *n* = 3 or 4 per group. Significance was determined at *P *< 0.05, and different letters indicate statistical differences. All photographs were taken at 20× magnification. Scale bar: 25 µm.

Immunohisochemistry data include only group‐housed mice. In the liver, CD68 staining was significantly greater (*P *= 0.0001) in male Western/Binge mice compared with male Control. Both male Western (*P *= 0.028) and Control/Binge (*P *= 0.017) were also higher than male Control. In females, Western (*P *= 0.004) and Western/Binge (*P *= 0.008) groups both had greater staining area of CD68 than female Control. Female Western (*P *= 0.003) and Western/Binge (*P *= 0.007) groups also had greater staining than male Control. Female Control/Binge CD68 area was not significantly different from female Control, Western or Western/Binge. CD8 staining area was significantly greater in the male Western/Binge group compared with male Control (*P *< 0.0001), Control/Binge (*P *= 0.0019) and Western (*P *= 0.036) (Figure 6a). The male Western group had greater (*P *= 0.048) CD8 area than male Control as well. In females, Western (*P *= 0.017) and Western/Binge (*P *= 0.017) groups had greater CD8 staining compared with female Control (Figure 6b). Across sexes, male Western/Binge had higher CD8 than only female Control (*P *< 0.0001) and Control/Binge (*P *= 0.013). No significant differences in splenic immunohistochemical staining for CD68, CD8 or CD45 were noted (Figure 7).

## 4 DISCUSSION

This study suggests that a Western diet plays a more significant role in liver health than binge drinking, but its co‐consumption with binge drinking results in more progressive disease in males. Alcohol consumption increased overall caloric consumption, which aligns with studies in humans observing a bidirectional relationship between unhealthy food choices and alcohol consumption (Breslow et al., 2010; Scott et al., 2020) (Figure 1a). Sex hormones also play a major role in diet and drinking patterns, with higher testosterone being linked to increased calorie consumption and risk for alcohol‐use disorders and higher oestrogen linked to suppression of appetite and increased binge drinking (Erol et al., 2019; Hirschberg, 2012). Although we did not measure sex hormones in our mice, tracking of food and alcohol consumption indicated that Western and Western/Binge males ate more than all other groups and that Western diet resulted in increased binge drinking regardless of sex (Figure 1b,c). As a surrogate of sex hormones, we did collect and weigh testes, seminal vesicles, ovaries and uteri, but no differences were observed (Figure 1f–i).

Increases in caloric consumption did not translate into differences in final body, liver, spleen, gonadal adipose tissue or retroperitoneal adipose tissue weight between diet and binge drinking groups within sex, although Western diet regardless of binge drinking did result in significantly heavier livers in males (Figure 2a–e). This lack of weight difference between diet and binge drinking groups in males might be explained by binge drinking causing a reduction in FCR relative to respective diet‐alone groups (Figure 1d). Alcohol consumption, even in moderation, is known to disrupt nutrient metabolism, reducing absorption of amino acids, glucose and lipids (Butts et al., 2023). Curiously, female FCR was not impacted by binge drinking, with no differences observed between female diet and drinking groups (Figure 1d). Female Western/Binge mice did have a trend to be heavier than female Control mice, but the difference was not significant. Female mice have been shown to have some protection from diet‐induced liver disease, which might account for the lack of significant liver weight differences (Meyer et al., 2025). Although weight and consumption patterns are important indices of health, liver disease can persist in the absence of weight gain, and more sensitive measures of liver health and function are preferable for determining disease status (Eslam et al., 2022).

Clinically, analyses of ALT and AST are often used to assess potential liver diseases, including MASLD, MASH and alcohol‐associated liver diseases (Kwo et al., 2017) [Correction added on 7 March 2026, after first online publication: The phrase metabo MASI I has been corrected to read “MASH.”]. In our study, a clear separation between males and females emerged in response to Western diet and binge drinking in these measures. Male Western and Western/Binge groups had significantly increased ALT relative to male Control and Control/Binge, with Western/Binge having a significant increase in AST compared with all other male groups (Figure 5c,d). In females, no differences across all groups were observed for ALT, and only Western/Binge and Control groups were significantly different for AST (Figure 5c,d). It should be noted that AST levels were consistently high across all our groups in comparison to expected values. ALT is primarily expressed in the liver, whereas AST is expressed in other organs, such as the heart and skeletal muscle, potentially indicating that Western diet and binge drinking resulted in more global damage than either treatment alone, but further research would be needed to determine this potential (Cacciola et al., 2017). Although the ratio of AST:ALT can be used to gain a deeper understanding of disease, with higher ratios indicative of alcohol‐related liver disease and lower ratios indicative of MASLD or MASH, it has limited utility in our study owing to high measurements of ALT and AST being present in male Western and Western/Binge groups, driving ratios down (Figure 5e) (Nyblom et al., 2004; Sorbi et al., 1999).

Histology remains the gold standard in diagnosing liver disease (Kleiner et al., 2019). For males, Western/Binge animals had the most evidence of steatosis amongst males, with increased amounts of both micro‐ and macrovesicular steatosis (Figure 3; Table 2). The increase in microvesicular steatosis is biologically significant, because this type of steatosis is associated with worsening liver health outcomes and disease progression (Oleszczuk et al., 2007). Meanwhile, female Western mice had the most evidence of steatosis and macrovesicular steatosis, but Western/Binge had more evidence of microvesicular steatosis (Figure 3; Table 2). To expand these results, we performed a triglyceride assay and found that male Western and Western/Binge groups had the highest triglyceride content compared with all other groups (Figure 4a). Female Western and Western/Binge groups did show an increase in hepatic triglycerides that was non‐significant from male Western and Western/Binge, in addition to all Control and Control/Binge groups, regardless of sex (Figure 4a). We take this to mean that males experienced a more pronounced increase in hepatic triglycerides, but females also experienced an increase in response to unhealthy diet. These results highlight the importance of histology, because triglyceride measures alone did not differentiate the type of steatosis pattern, which can often be overlooked in management of MASLD when less precise diagnostic measures are used (Bozic et al., 2022).

Sex‐specific differences in disease persisted when we analysed inflammation, oxidative stress and immune response, all of which are key factors in the progression of MASLD to MASH (Y. Li et al., 2024). In histological evaluation, male Western/Binge and female Western groups were more likely to have lobular inflammation than other groups amongst their sex, indicating progression to steatohepatitis (Figure 3; Table 2). Liver lipid peroxidation and antioxidant levels were measured to determine whether a disruption in pro‐ and antioxidant balance was driving inflammation (Arroyave‐Ospina et al., 2021). In males, although lipid peroxidation was significantly increased in Western and Western/Binge groups, no differences in antioxidants were observed within sex (Figure 4b–d). In females, again Western and Western/Binge groups both had increased lipid peroxidation, but only female Western had a significant reduction in hepatic SOD compared with all male groups, female Control and female Control/Binge (Figure 4b–d). Imbalance in oxidative stress is a known contributor to the progression of liver disease, but typically males experience increased pro‐oxidative substances and reduced antioxidant capacity compared with females (Lonardo et al., 2019). Although reductions in SOD in female mice with liver disease are not typically reported, progression to steatohepatitis and chronic alcohol consumption have both been shown to decrease SOD expression and effectiveness (Gopal et al., 2025; Svobodová et al., 2025).

Given that immune activation and disruption are key to disease progression, hepatic MPO activity was measured as a rough assessment of neutrophil infiltration, and again sex differences were observed (Koop et al., 2020). Only in females, MPO activity was significantly reduced in Western/Binge compared with Control, with no significant differences reported amongst the other groups (Figure 4e). Although low MPO activity can be associated with negative health outcomes, MPO reduction in mice attenuates liver disease progression, which might explain, in part why female Western/Binge mice appeared to have marginal health improvements compared with Western (Koop et al., 2020; Rensen et al., 2012). Immunohistochemical staining for hepatic CD68 showed all male experimental groups to have an increase compared with male Control, and only female Western and Western/Binge groups were significantly different from female Control (Figure 6a,b). CD68 is an important clinical marker for macrophages, or Kupffer cells in the liver, which play a key role in clearing debris and aiding in healing (Kolios et al., 2006). In progression of liver disease, Kupffer cells release various inflammatory mediators, which are associated with transition to MASH and fibrosis (Wenfeng et al., 2015). Immunohistochemical staining for CD8, however, showed male Western/Binge mice to have significantly higher amounts than all other male groups, and female Western and Western/Binge to have increased CD8 compared with female Control (Figure 6b). CD8^+^ T cells play a key role in response to disease, and they can activate hepatic stellate cells, leading to fibrosis (Koda et al., 2021). The elevation of CD8 compared with sex‐matched Control in all experimental groups suggests that both unhealthy diet and binge drinking act to increase CD8. Although this is associated with fibrosis, we did not observe any evidence of fibrosis in our histology. Fibrosis in mouse models is variable, and diet‐induced models of disease typically take >20 weeks, making it unsurprising that our study failed to reach the level of fibrosis (Wei et al., 2020). Serum globulin levels continued the pattern of male Western/Binge and female Western having higher values than their respective Control, further indicating pro‐inflammatory response (Figure 5f), but splenic analysis of CD45, CD68 and CD8 did not reveal any differences (H. Wang et al., 2016) (Figure 7).

Although binge drinking on its own did not result in overt evidence of liver damage, a few measures suggest that it did make an impact. The Western/Binge groups, regardless of sex, demonstrated a suppression of total cholesterol (Figure 5b). Moderate drinking has been shown to lower cholesterol; however, binge drinking is not associated with improvements of cholesterol, calling into question whether the less intense drinking protocol in the present study was more akin to moderate amounts of alcohol consumption (Rosoff et al., 2019; Suzuki et al., 2025). However, male Control/Binge mice had increased fasting blood glucose compared with female Control/Binge, Western and Western/Binge, which mirrors data that heavy drinking increases fasting glucose (Figure 5a) (Leggio et al., 2009). In females, Control/Binge mice had an increase in serum AST that was non‐significantly different from male Western/Binge mice, which had the highest AST levels (Figure 5d). Females are susceptible to alcohol‐related damage, and high‐intensity binge drinking has been shown to cause increases in AST (Rosoff et al., 2019). Male and female Control/Binge mice also both experienced an increase in liver CD68 and CD8 that was non‐significantly different from male Western/Binge and female Western groups, which aligns with previous research demonstrating that even a single bout of binge drinking can increase immune cell activation (Figure 6a,b) (Stadlbauer et al., 2019). Taken together, the binge drinking in the present study had a modest effect on health, but more research on the hepatic impacts of binge drinking is needed to gain a better understanding of its impacts (D'Alessandro et al., 2023).

The differential response between males and females highlights the importance of exploring the role of sex in liver disease, which is largely understudied (Ji & Cheng, 2023). We are, to the best of our knowledge, the first group to look at how the combination of a Western diet and binge drinking impact hepatic health in females. Here, we show that Western diet alone damages the liver more than co‐consumption with binge drinking in females. This is not to say that binge drinking paired with a Western diet promotes health, because female Western/Binge mice showed severe steatosis and evidence of steatohepatitis. In observing responses to diet and binge drinking, we observed male mice to have alterations to measures associated with lipid metabolism, which is supported by previous research (Nagral et al., 2022; Varlamov et al., 2015). Meanwhile, suppression of the antioxidant system was more common in females, which is not commonly reported. A Western diet is known to suppress the antioxidant system (Bhol et al., 2024; Chen et al., 2020), but why females showed this shift is not clear, especially considering that females typically have more robust antioxidant responses than males (Al‐Gubory & Garrel, 2016). Lastly, male Western/Binge did show increased evidence of liver disease progression in histology and several key measures. Although these differences were not as overt as other groups have reported (Babuta et al., 2024; Buyco et al., 2023), they do suggest that addition of binge drinking to an unhealthy diet worsens liver health.

Our study is not without limitations. We altered the standard Drinking in the Dark protocol to include fewer days of binge drinking and more time between binge drinking bouts (Thiele & Navarro, 2014). This was intentional, because anecdotally we observed that college students at our institution typically binge drinking 3 days per week with breaks between binge bouts, which is similar to reported findings on the irregular patterns of college student drinking (Hoeppner et al., 2012; Krieger et al., 2018). The comparatively lower amount of drinking time might make our results difficult to compare with other studies that have used a heavier drinking pattern. We did not measure blood alcohol concentration, which would have been helpful in determining the level of intoxication in the mice. Furthermore, use of metabolic cages would have allowed for more precise measurement of intakes and outputs, such as faeces and urine. The initial use of single‐housed animals allowed us to assess the amount of alcohol and food being consumed, but future studies will measure blood alcohol content for more precise measures. Our follow‐up cohort was co‐housed, introducing potential for differences, because housing density has been documented to alter results in rodent experiments (Laprade, 2023; Võikar et al., 2005). To remedy this issue, we performed statistical analyses on single‐ and group‐housed animals for all measures that included data from both sets to confirm that no differences existed (Table S1). As stated above, hormones play a major role in eating and drinking behaviours and liver disease onset. Future studies will disentangle the role of sex hormones in response to combined binge drinking and unhealthy diet. Lastly, advanced liver disease, such as fibrosis, takes a substantial amount of time to develop, and 12 weeks was not sufficient to see this progression. However, our 12 week study provides foundational evidence of earlier changes in response to unhealthy diet and binge drinking. Even with these limitations, this study, to our knowledge, is the first to explore sex differences with respect to a Western diet and binge drinking on the liver.

## 5 CONCLUSION

Here, we show that diet and sex are major drivers in liver disease progression, with binge drinking playing a smaller, but not insignificant role. Future studies should seek to gain a deeper understanding of the molecular mechanisms at play and consider a longer study time frame to explore whether progression of liver disease continues with time.

## AUTHOR CONTRIBUTIONS

This study was performed in R. Chris Skinner's and Matthew A. Caporizzo's laboratories. R. Chris Skinner, Matthew A. Caporizzo, and Ihsan Shawki Akili conceived and designed this research. Ihsan Shawki Akili, Corina Miko, Azhar Saeed, Colby Filosa, Madeline Orlowski, Alanna Sherman, Aldi Chan, Madison Wan, Jake Grenon, Alec Thibeault, Catherine Young, Melinda Wetzel, Virginia Clubb, and R. Chris Skinner performed experiments. Ihsan Shawki Akili, Corina Miko, Azhar Saeed, Colby Filosa, Madeline Orlowski, Alanna Sherman, Aldi Chan, Jake Grenon, R. Chris Skinner analyzed data and interpreted results of experiments. Ihsan Shawki Akili, Corina Miko, Colby Filosa, Madeline Orlowski, Alanna Sherman and R. Chris Skinner prepared the figures. Ihsan Shawki Akili, Corina Miko, and R. Chris Skinner drafted the manuscript. All authors edited and revised the manuscript. All authors have reviewed the final version of the manuscript and agree to be accountable for all aspects of the work in ensuring that questions related to accuracy or integrity of any part of the work are approrpriately investigated and resolved. All persons designated as authors qualify for authorship, and all those who qualify for authorship are listed. During the preparation of this work the authors did not use any AI technologies in the writing process.

## CONFLICT OF INTEREST

None declared.

## Supporting information




**Table S1**. Data comparing single and co‐housed male mice.
**Table S2**. Data comparing single and co‐housed female mice.

## Data Availability

All data are available in the manuscript. All raw data is available upon request to the corresponding author.

## References

[eph70233-bib-0001] Akkız, H. , Gieseler, R. K. , & Canbay, A. (2024). Liver fibrosis: From basic science towards clinical progress, focusing on the central role of hepatic stellate cells. International Journal of Molecular Sciences, 25(14), 7873. 39063116 10.3390/ijms25147873 PMC11277292

[eph70233-bib-0002] Al‐Gubory, K. H. , & Garrel, C. (2016). Sex‐specific divergence of antioxidant pathways in fetal brain, liver, and skeletal muscles. Free Radical Research, 50(3), 366–373. 26765668 10.3109/10715762.2015.1130224

[eph70233-bib-0003] Ameer, F. , Scandiuzzi, L. , Hasnain, S. , Kalbacher, H. , & Zaidi, N. (2014). De novo lipogenesis in health and disease. Metabolism, 63(7), 895–902. 24814684 10.1016/j.metabol.2014.04.003

[eph70233-bib-0004] Arroyave‐Ospina, J. C. , Wu, Z. , Geng, Y. , & Moshage, H. (2021). Role of oxidative stress in the pathogenesis of non‐alcoholic fatty liver disease: Implications for prevention and therapy. Antioxidants, 10(2), 174. 33530432 10.3390/antiox10020174 PMC7911109

[eph70233-bib-0005] Babuta, M. , Morel, C. , de Carvalho Ribeiro, M. , Datta, A. A. , Calenda, C. , Copeland, C. , Nasser, I. , & Szabo, G. (2024). A novel experimental model of MetALD in male mice recapitulates key features of severe alcohol‐associated hepatitis. Hepatology Communications, 8(7), e0450. 38896082 10.1097/HC9.0000000000000450 PMC11186819

[eph70233-bib-0006] Ballestri, S. , Nascimbeni, F. , Baldelli, E. , Marrazzo, A. , Romagnoli, D. , & Lonardo, A. (2017). NAFLD as a sexual dimorphic disease: Role of gender and reproductive status in the development and progression of nonalcoholic fatty liver disease and inherent cardiovascular risk. Advances in Therapy, 34(6), 1291–1326. 28526997 10.1007/s12325-017-0556-1 PMC5487879

[eph70233-bib-0007] Bhol, N. K. , Bhanjadeo, M. M. , Singh, A. K. , Dash, U. C. , Ojha, R. R. , Majhi, S. , Duttaroy, A. K. , & Jena, A. B. (2024). The interplay between cytokines, inflammation, and antioxidants: Mechanistic insights and therapeutic potentials of various antioxidants and anti‐cytokine compounds. Biomedicine & Pharmacotherapy, 178, 117177. 39053423 10.1016/j.biopha.2024.117177

[eph70233-bib-0008] Bohm, M. K. (2021). Binge drinking among adults, by select characteristics and state—United States, 2018. MMWR. Morbidity and Mortality Weekly Report, 70(41), 1441–1446. 34648484 10.15585/mmwr.mm7041a2 PMC8631283

[eph70233-bib-0009] Bozic, D. , Podrug, K. , Mikolasevic, I. , & Grgurevic, I. (2022). Ultrasound methods for the assessment of liver steatosis: A critical appraisal. Diagnostics, 12(10), 2287. 36291976 10.3390/diagnostics12102287 PMC9600709

[eph70233-bib-0010] Breslow, R. A. , Guenther, P. M. , Juan, W. , & Graubard, B. I. (2010). Alcoholic beverage consumption, nutrient intakes, and diet quality in the US adult population, 1999–2006. Journal of the American Dietetic Association, 110(4), 551–562. 20338281 10.1016/j.jada.2009.12.026 PMC2864068

[eph70233-bib-0011] Butts, M. , Sundaram, V. L. , Murughiyan, U. , Borthakur, A. , & Singh, S. (2023). The influence of alcohol consumption on intestinal nutrient absorption: A comprehensive review. Nutrients, 15(7), 1571. 37049411 10.3390/nu15071571 PMC10096942

[eph70233-bib-0012] Buyco, D. G. , Dempsey, J. L. , Scorletti, E. , Jeon, S. , Lin, C. , Harkin, J. , Bayen, S. , Furth, E. E. , Martin, J. , Delima, M. , Hooks, R. , Sostre‐Colón, J. , Gharib, S. A. , Titchenell, P. M. , & Carr, R. M. (2023). Concomitant western diet and chronic‐binge alcohol dysregulate hepatic metabolism. PLoS ONE, 18(5), e0281954. 37134024 10.1371/journal.pone.0281954 PMC10155975

[eph70233-bib-0013] Cacciola, I. , Scoglio, R. , Alibrandi, A. , Squadrito, G. , Raimondo, G. , Alibrando, A. , Amato, S. , Crescenti, A. , Crescenti, F. , Di Geronimo, L. , Inferrera, S. , La Malfa, L. , Maneri, G. , Marino, S. , Pernice, M. , Saccà, F. , Saija, G. , & Salanitro, L. , & SIMG‐Messina Hypertransaminasemia Study Group . (2017). Evaluation of liver enzyme levels and identification of asymptomatic liver disease patients in primary care. Internal and Emergency Medicine, 12(2), 181–186. 27644706 10.1007/s11739-016-1535-2

[eph70233-bib-0014] Chen, Z. , Tian, R. , She, Z. , Cai, J. , & Li, H. (2020). Role of oxidative stress in the pathogenesis of nonalcoholic fatty liver disease. Free Radical Biology & Medicine, 152, 116–141. 32156524 10.1016/j.freeradbiomed.2020.02.025

[eph70233-bib-0015] D'Alessandro, S. , Carter, L. , & Webster, C. (2023). Binge drinking: A review and research agenda. Journal of Consumer Behaviour, 22(1), 177–198.

[eph70233-bib-0016] Duan, Y. , Pan, X. , Luo, J. , Xiao, X. , Li, J. , Bestman, P. L. , & Luo, M. (2022). Association of inflammatory cytokines with non‐alcoholic fatty liver disease. Frontiers in Immunology, 13, 880298. 35603224 10.3389/fimmu.2022.880298 PMC9122097

[eph70233-bib-0017] Erol, A. , Ho, A. M.‐C. , Winham, S. J. , & Karpyak, V. M. (2019). Sex hormones in alcohol consumption: A systematic review of evidence. Addiction Biology, 24(2), 157–169. 29280252 10.1111/adb.12589 PMC6585852

[eph70233-bib-0018] Eslam, M. , El‐Serag, H. B. , Francque, S. , Sarin, S. K. , Wei, L. , Bugianesi, E. , & George, J. (2022). Metabolic (dysfunction)‐associated fatty liver disease in individuals of normal weight. Nature Reviews Gastroenterology & Hepatology, 19(10), 638–651. 35710982 10.1038/s41575-022-00635-5

[eph70233-bib-0019] Gopal, T. , John Kathiravan, A. D. , Kabanov, A. V. , Casey, C. A. , & Saraswathi, V. (2025). The pathophysiology of alcohol‐associated liver disease: Focusing on superoxide dismutase 1 as a therapeutic target. Biology, 14(10), 1319. 41154722 10.3390/biology14101319 PMC12562268

[eph70233-bib-0020] Hagström, H. (2017). Alcohol consumption in concomitant liver disease: How much is too much? Current Hepatology Reports, 16(2), 152–157. 28706775 10.1007/s11901-017-0343-0 PMC5486588

[eph70233-bib-0021] Hirschberg, A. L. (2012). Sex hormones, appetite and eating behaviour in women. Maturitas, 71(3), 248–256. 22281161 10.1016/j.maturitas.2011.12.016

[eph70233-bib-0022] Hoeppner, B. B. , Barnett, N. P. , Jackson, K. M. , Colby, S. M. , Kahler, C. W. , Monti, P. M. , Read, J. , Tevyaw, T. , Wood, M. , Corriveau, D. , & Fingeret, A. (2012). Daily college student drinking patterns across the first year of college. Journal of Studies on Alcohol and Drugs, 73(4), 613–624. 22630800 10.15288/jsad.2012.73.613 PMC3364328

[eph70233-bib-0023] Huang, M. , Chen, H. , Wang, H. , Zhang, Y. , Li, L. , Lan, Y. , & Ma, L. (2025). Global burden and risk factors of MASLD: Trends from 1990 to 2021 and predictions to 2030. Internal and Emergency Medicine, 20(4), 1013–1024. 40019669 10.1007/s11739-025-03895-6 PMC12130103

[eph70233-bib-0024] Ji, H. , & Cheng, S. (2023). Sex differences in prevalence and prognosis of steatotic liver disease phenotypes: Biological sex matters. Journal of Hepatology, 80(2), e68–e69. 37619929 10.1016/j.jhep.2023.08.013 PMC10873108

[eph70233-bib-0025] Kanny, D. , Naimi, T. S. , Liu, Y. , & Brewer, R. D. (2020). Trends in total binge drinks per adult who reported binge drinking—United States, 2011–2017. Morbidity and Mortality Weekly Report, 69(2), 30–34. 31945030 10.15585/mmwr.mm6902a2 PMC6973354

[eph70233-bib-0026] Kleiner, D. E. , Brunt, E. M. , Wilson, L. A. , Behling, C. , Guy, C. , Contos, M. , Cummings, O. , Yeh, M. , Gill, R. , Chalasani, N. , Neuschwander‐Tetri, B. A. , Diehl, A. M. , Dasarathy, S. , Terrault, N. , Kowdley, K. , Loomba, R. , Belt, P. , Tonascia, J. , Lavine, J. E. , … for the Nonalcoholic Steatohepatitis Clinical Research Network . (2019). Association of histologic disease activity with progression of nonalcoholic fatty liver disease. Journal of the American Medical Association Network Open, 2(10), e1912565. 10.1001/jamanetworkopen.2019.12565 PMC6784786 31584681

[eph70233-bib-0027] Koda, Y. , Teratani, T. , Chu, P.‐S. , Hagihara, Y. , Mikami, Y. , Harada, Y. , Tsujikawa, H. , Miyamoto, K. , Suzuki, T. , Taniki, N. , Sujino, T. , Sakamoto, M. , Kanai, T. , & Nakamoto, N. (2021). CD8+ tissue‐resident memory T cells promote liver fibrosis resolution by inducing apoptosis of hepatic stellate cells. Nature Communications, 12(1), 4474. 10.1038/s41467-021-24734-0 PMC8298513 34294714

[eph70233-bib-0028] Kolios, G. , Valatas, V. , & Kouroumalis, E. (2006). Role of Kupffer cells in the pathogenesis of liver disease. World Journal of Gastroenterology : WJG, 12(46), 7413. 17167827 10.3748/wjg.v12.i46.7413 PMC4087584

[eph70233-bib-0029] Koop, A. C. , Thiele, N. D. , Steins, D. , Michaëlsson, E. , Wehmeyer, M. , Scheja, L. , Steglich, B. , Huber, S. , Schulze Zur Wiesch, J. , Lohse, A. W. , Heeren, J. , & Kluwe, J. (2020). Therapeutic targeting of myeloperoxidase attenuates NASH in mice. Hepatology Communications, 4(10), 1441–1458. 33024915 10.1002/hep4.1566 PMC7527691

[eph70233-bib-0030] Krieger, H. , Young, C. M. , Anthenien, A. M. , & Neighbors, C. (2018). The epidemiology of binge drinking among college‐age individuals in the United States. Alcohol Research: Current Reviews, 39(1), 23–30. 30557145 10.35946/arcr.v39.1.05 PMC6104967

[eph70233-bib-0031] Kwo, P. Y. , Cohen, S. M. , & Lim, J. K. (2017). ACG clinical guideline: Evaluation of abnormal liver chemistries. The American Journal of Gastroenterology, 112(1), 18–35. 27995906 10.1038/ajg.2016.517

[eph70233-bib-0032] Laprade, K. (2023). Housing effect on alcohol consumption in a binge drinking model following foot shock stress. Graduate College Dissertations and Theses. https://scholarworks.uvm.edu/graddis/1749

[eph70233-bib-0033] Leggio, L. , Ray, L. A. , Kenna, G. A. , & Swift, R. M. (2009). Blood glucose level, alcohol heavy drinking and alcohol craving during treatment for alcohol dependence: Results from the Combined Pharmacotherapies and Behavioral Interventions for Alcohol Dependence (COMBINE) Study. Alcohol, Clinical and Experimental Research, 33(9), 1539–1544. 10.1111/j.1530-0277.2009.00982.x PMC2955866 19485973

[eph70233-bib-0034] Li, J. , Zhou, J. , Li, P. , Wang, Y. , Ridderhof, N. , Al‐Tawfiq, J. A. , Brouwer, W. P. , Chen, K. , de Knegt, R. J. , Peppelenbosch, M. P. , Hansen, B. E. , Engel, M. F. M. , Zheng, M.‐H. , Memish, Z. A. , Eslam, M. , Janssen, H. L. A. , Pan, Q. , & Ayada, I. (2025). The global prevalence and impact of steatotic liver disease and viral infections: A systematic review and meta‐analysis. Hepatology Communications, 9(5), e0689. 40227096 10.1097/HC9.0000000000000689 PMC11999411

[eph70233-bib-0035] Li, Y. , Yang, P. , Ye, J. , Xu, Q. , Wu, J. , & Wang, Y. (2024). Updated mechanisms of MASLD pathogenesis. Lipids in Health and Disease, 23(1), 117. 38649999 10.1186/s12944-024-02108-x PMC11034170

[eph70233-bib-0036] Liu, J. , Li, C. , Yang, Y. , Li, J. , Sun, X. , Zhang, Y. , Liu, R. , Chen, F. , & Li, X. (2025). Special correlation between diet and MASLD: Positive or negative? Cell & Bioscience, 15(1), 44. 40221799 10.1186/s13578-025-01382-1 PMC11992798

[eph70233-bib-0037] Lonardo, A. , Nascimbeni, F. , Ballestri, S. , Fairweather, D. , Win, S. , Than, T. A. , Abdelmalek, M. F. , & Suzuki, A. (2019). Sex differences in nonalcoholic fatty liver disease: State of the art and identification of research gaps. Hepatology, 70(4), 1457–1469. 30924946 10.1002/hep.30626 PMC6766425

[eph70233-bib-0038] Meyer, J. , Teixeira, A. M. , Richter, S. , Larner, D. P. , Syed, A. , Klöting, N. , Matz‐Soja, M. , Gaul, S. , Barnikol‐Oettler, A. , Kiess, W. , Le Duc, D. , Penke, M. , & Garten, A. (2025). Sex differences in diet‐induced MASLD—Are female mice naturally protected? Frontiers in Endocrinology, 16, 1567573. 40162312 10.3389/fendo.2025.1567573 PMC11949793

[eph70233-bib-0039] Nagral, A. , Bangar, M. , Menezes, S. , Bhatia, S. , Butt, N. , Ghosh, J. , Manchanayake, J. H. , Mahtab, M. A. , & Singh, S. P. (2022). Gender differences in nonalcoholic fatty liver disease. Euroasian Journal of Hepato‐Gastroenterology, 12(S1), S19–S25. 36466099 10.5005/jp-journals-10018-1370 PMC9681575

[eph70233-bib-0040] Nyblom, H. , Berggren, U. , Balldin, J. , & Olsson, R. (2004). HIgh AST/ALT ratio may indicate advanced alcoholic liver disease rather than heavy drinking. Alcohol and Alcoholism, 39(4), 336–339. 15208167 10.1093/alcalc/agh074

[eph70233-bib-0041] Oleszczuk, A. , Spannbauer, M. , Tannapfel, A. , Blüher, M. , Hengstler, J. , Pietsch, U.‐C. , Schuhmacher, A. , Wittekind, C. , Hauss, J. P. , & Schön, M. R. (2007). Regenerative capacity differs between micro‐ and macrovesicular hepatic steatosis. Experimental and Toxicologic Pathology, 59(3‐4), 205–213. 17869075 10.1016/j.etp.2007.05.009

[eph70233-bib-0042] Osna, N. A. , Rasineni, K. , Ganesan, M. , Donohue, T. M. , & Kharbanda, K. K. (2022). Pathogenesis of alcohol‐associated liver disease. Journal of Clinical and Experimental Hepatology, 12(6), 1492–1513. 36340300 10.1016/j.jceh.2022.05.004 PMC9630031

[eph70233-bib-0043] Rafaqat, S. , Gluscevic, S. , Mercantepe, F. , Rafaqat, S. , & Klisic, A. (2024). Interleukins: Pathogenesis in non‐alcoholic fatty liver disease. Metabolites, 14(3), 153. 38535313 10.3390/metabo14030153 PMC10972081

[eph70233-bib-0044] Rensen, S. S. , Bieghs, V. , Xanthoulea, S. , Arfianti, E. , Bakker, J. A. , Shiri‐Sverdlov, R. , Hofker, M. H. , Greve, J. W. , & Buurman, W. A. (2012). Neutrophil‐derived myeloperoxidase aggravates non‐alcoholic steatohepatitis in low‐density lipoprotein receptor‐deficient mice. PLoS ONE, 7(12), e52411. 23285030 10.1371/journal.pone.0052411 PMC3527496

[eph70233-bib-0045] Rosoff, D. B. , Charlet, K. , Jung, J. , Lee, J. , Muench, C. , Luo, A. , Longley, M. , Mauro, K. L. , & Lohoff, F. W. (2019). Association of high‐intensity binge drinking with lipid and liver function enzyme levels. Journal of the American Medical Association Network Open, 2(6), e195844. 10.1001/jamanetworkopen.2019.5844 PMC6575145 31199452

[eph70233-bib-0046] Scott, S. , Muir, C. , Stead, M. , Fitzgerald, N. , Kaner, E. , Bradley, J. , Wrieden, W. , Power, C. , & Adamson, A. (2020). Exploring the links between unhealthy eating behaviour and heavy alcohol use in the social, emotional and cultural lives of young adults (aged 18–25): A qualitative research study. Appetite, 144, 104449. 31520670 10.1016/j.appet.2019.104449

[eph70233-bib-0047] Seidemann, L. , Lippold, C. P. , Rohm, C. M. , Eckel, J. C. , Schicht, G. , Matz‐Soja, M. , Berg, T. , Seehofer, D. , & Damm, G. (2024). Sex hormones differently regulate lipid metabolism genes in primary human hepatocytes. BioMed Central Endocrine Disorders, 24(1), 135. 39090659 10.1186/s12902-024-01663-9 PMC11292922

[eph70233-bib-0048] Sorbi, D. , Boynton, J. , & Lindor, K. D. (1999). The ratio of aspartate aminotransferase to alanine aminotransferase: Potential value in differentiating nonalcoholic steatohepatitis from alcoholic liver disease. Official Journal of the American College of Gastroenterology | ACG, 94(4), 1018–1022. 10.1111/j.1572-0241.1999.01006.x 10201476

[eph70233-bib-0049] Stadlbauer, V. , Horvath, A. , Komarova, I. , Schmerboeck, B. , Feldbacher, N. , Wurm, S. , Klymiuk, I. , Durdevic, M. , Rainer, F. , Blesl, A. , Stryeck, S. , Madl, T. , Stiegler, P. , & Leber, B. (2019). A single alcohol binge impacts on neutrophil function without changes in gut barrier function and gut microbiome composition in healthy volunteers. PLoS ONE, 14(2), e0211703. 30707717 10.1371/journal.pone.0211703 PMC6358085

[eph70233-bib-0050] Suzuki, T. , Fukui, S. , Shinozaki, T. , Asano, T. , Yoshida, T. , Aoki, J. , & Mizuno, A. (2025). Lipid profiles after changes in alcohol consumption among adults undergoing annual checkups. Journal of the American Medical Association Network Open, 8(3), e250583. 10.1001/jamanetworkopen.2025.0583 PMC11904732 40072433

[eph70233-bib-0051] Svobodová, G. , Horní, M. , Velecká, E. , & Boušová, I. (2025). Metabolic dysfunction‐associated steatotic liver disease‐induced changes in the antioxidant system: A review. Archives of Toxicology, 99(1), 1–22. 39443317 10.1007/s00204-024-03889-x PMC11748479

[eph70233-bib-0052] Thiele, T. E. , & Navarro, M. (2014). “Drinking in the Dark” (DID) procedures: A model of binge‐like ethanol drinking in non‐dependent mice. Alcohol (Fayetteville, N.Y.), 48(3), 235–241. 24275142 10.1016/j.alcohol.2013.08.005 PMC4004717

[eph70233-bib-0053] Understanding Binge Drinking | National Institute on Alcohol Abuse and Alcoholism (NIAAA) . (n.d.). Retrieved January 21, 2025, from https://www.niaaa.nih.gov/publications/brochures‐and‐fact‐sheets/binge‐drinking

[eph70233-bib-0054] Varlamov, O. , Bethea, C. L. , & Roberts, C. T. (2015). Sex‐specific differences in lipid and glucose metabolism. Frontiers in Endocrinology, 5, 241. 25646091 10.3389/fendo.2014.00241 PMC4298229

[eph70233-bib-0055] Võikar, V. , Polus, A. , Vasar, E. , & Rauvala, H. (2005). Long‐term individual housing in C57BL/6J and DBA/2 mice: Assessment of behavioral consequences. Genes, Brain and Behavior, 4(4), 240–252. 15924556 10.1111/j.1601-183X.2004.00106.x

[eph70233-bib-0056] Wang, H. , Xu, H. , Qu, L. , Wang, X. , Wu, R. , Gao, X. , Jin, Q. , & Niu, J. (2016). Red blood cell distribution width and globulin, noninvasive indicators of fibrosis and inflammation in chronic hepatitis patients. European Journal of Gastroenterology & Hepatology, 28(9), 997–1002. 27167453 10.1097/MEG.0000000000000662

[eph70233-bib-0057] Wang, H. J. , Gao, B. , Zakhari, S. , & Nagy, L. E. (2012). Inflammation in alcoholic liver disease. Annual Review of Nutrition, 32(1), 343–368. 10.1146/annurev-nutr-072610-145138 PMC3670145 22524187

[eph70233-bib-0058] Wei, G. , An, P. , Vaid, K. A. , Nasser, I. , Huang, P. , Tan, L. , Zhao, S. , Schuppan, D. , & Popov, Y. V. (2020). Comparison of murine steatohepatitis models identifies a dietary intervention with robust fibrosis, ductular reaction, and rapid progression to cirrhosis and cancer. American Journal of Physiology‐Gastrointestinal and Liver Physiology, 318(1), G174–G188. 31630534 10.1152/ajpgi.00041.2019 PMC6985845

[eph70233-bib-0059] Wenfeng, Z. , Wu, Y. , Di, M. , Gong, J. , Chuanxin, W. , & Chun, H. (2015). Kupffer cells: Increasingly significant role in nonalcoholic fatty liver disease. Annals of Hepatology, 13(5), 489–495. 25152980

[eph70233-bib-0060] Yang, T. , Yin, J. , Li, J. , & Wang, Q. (2024). The influence of different combinations of cardiometabolic risk factors on the prevalence of MASLD and risk of advanced fibrosis deserves attention. Journal of Hepatology, 80(2), e82–e85. 37813244 10.1016/j.jhep.2023.09.030

