# Supplementary material for: The impact of a Western diet and binge drinking on metabolic dysfunction‐associated steatotic liver disease in male and female mice
Source: Exp Physiol. 2026 Feb 17;111(4):1861–74. doi: 10.1113/EP093502 (PMC13140200; doi:10.1113/EP093502)
Supplement: Supplementary file 1 — Table S1. Data comparing single and co‐housed male mice. Table S2. Data comparing single and co‐housed female mice. [file EPH-111-1861-s001.docx]

**Supplemental Tables**

| **Supplemental Table 1.** Data comparing single and co-housed male mice. | | | | | | | | |
| --- | --- | --- | --- | --- | --- | --- | --- | --- |
|  | Single | | | | Group | | | |
| **Measure** | **Control** | **Control/Binge** | **Western** | **Western/Binge** | **Control** | **Control/Binge** | **Western** | **Western/Binge** |
| **Final** | 31.80 ± 2^b^ | 29.80 ± 1^b^ | 44.93 ± 9^a^ | 43.95 ± 11^a^ | 32.13 ± 2^b^ | 31.83 ± 1^b^ | 45.08 ± 12^a^ | 44.25 ±8^a^ |
| **Liver** | 1.13 ± 0.4^b^ | 1.11 ± 0.2^b^ | 2.50 ± 1^a^ | 2.48 ± 1^a^ | 1.09 ± 0.06^a,c^ | 1.32 ± 0.04^a,c^ | 2.39 ± 0.5^a,b^ | 2.4 ± 0.75^a,c^ |
| **Spleen** | 0.08 ± 0.01^a,c^ | 0.08 ± 0.^a,c^ | 0.10 ± 0.05^a^ | 0.09 ± 0.04^a,b^ | 0.06 ± 0.01b,c | 0.06 ± 0.01^c^ | 0.08 ± 0.03^a,c^ | 0.10 ± 0.02^a^ |

Significance was determined at *p*<0.05 and different letters indicate statistical differences with n=3-7/group.

| **Supplemental Table 2.** Data comparing single and co-housed female mice. | | | | | | | | |
| --- | --- | --- | --- | --- | --- | --- | --- | --- |
|  | Single | | | | Group | | | |
| **Measure** | **Control** | **Control/Binge** | **Western** | **Western/Binge** | **Control** | **Western** | **Western/Binge** | **Western** |
| **Final** | 22.02 ± 3^b^ | 21.48 ± 2^b^ | 30.26 ± 6^a^ | 32.04 ± 7^a^ | 21.825 ± 1^b^ | 22.38 ± 2^b^ | 30.87 ± 9^a^ | 33.83 ±8^a^ |
| **Liver** | 0.77 ± 0.05^d^ | 0.83 ± 0.1^c,d^ | 1.21 ± 0.25^a,b^ | 1.41 ± 0.25^a^ | 0.84 ± 0.05^b,d^ | 0.91 ± 0.01^b,d^ | 1.15 ± 0.25^a,b,c^ | 1.29 ± 0.30^a,b^ |
| **Spleen** | 0.08 ± 0.01^a^ | 0.09 ± 0.02^a^ | 0.12 ± 0.03^a^ | 0.12 ± 0.05^a^ | 0.10 ± 0.03^a^ | 0.11 ± 0.01^a^ | 0.09 ± 0.04^a^ | 0.12 ± 0.06^a^ |

Significance was determined at *p*<0.05 and different letters indicate statistical differences with n=3-7/group.
